# Structure and function predictions of the Msa protein in *Staphylococcus aureus*

**DOI:** 10.1186/1471-2105-8-S7-S5

**Published:** 2007-11-01

**Authors:** Vijayaraj Nagarajan, Mohamed O Elasri

**Affiliations:** 1 Department of Biological Sciences, The University of Southern Mississippi, Hattiesburg, MS, 39406, USA

## Abstract

**Background:**

*Staphylococcus aureus *is a human pathogen that causes a wide variety of life-threatening infections using a large number of virulence factors. One of the major global regulators used by *S. aureus *is the staphylococcal accessory regulator (*sarA*). We have identified and characterized a new gene (modulator of *sarA*: *msa*) that modulates the expression of *sarA*. Genetic and functional analysis shows that *msa *has a global effect on gene expression in *S. aureus*. However, the mechanism of Msa function is still unknown. Function predictions of Msa are complicated by the fact that it does not have a homologous partner in any other organism. This work aims at predicting the structure and function of the Msa protein.

**Results:**

Preliminary sequence analysis showed that Msa is a putative membrane protein. It would therefore be very difficult to purify and crystallize Msa in order to acquire structure information about this protein. We have used several computational tools to predict the physico-chemical properties, secondary structural features, topology, 3D tertiary structure, binding sites, motifs/patterns/domains and cellular location. We have built a consensus that is derived from analysis using different algorithms to predict several structural features. We confirm that Msa is a putative membrane protein with three transmembrane regions. We also predict that Msa has phosphorylation sites and binding sites suggesting functions in signal transduction.

**Conclusion:**

Based on our predictions we hypothesise that Msa is a novel signal transducer that might be involved in the interaction of the *S. aureus *with its environment.

## Background

### Introduction

*Staphylococcus aureus *is an important human pathogen that causes several diseases ranging from superficial skin infections to life-threatening diseases such as osteomyelitis and endocarditis. *S. aureus *is capable of infecting a wide range of tissues in humans because of the large number of virulence factors and the complex regulatory networks that control them [1]. In addition, *S. aureus *is increasingly resistant to multiple antibiotics thus becoming a growing threat to public health. There is an urgent need to understand the complex regulatory networks used by *S. aureus *to cause disease. Regulatory networks are attractive therapeutic targets for future treatment of antibiotic resistant infections.

### Modulator of *sarA *(*msa*)

One of the important global regulators of virulence in *S. aureus *is the Staphylococcal accessory regulator (*sarA*) [2]. *sarA *regulates over 100 genes in *S. aureus *several of which are associated with virulence [3]. *sarA *plays an important role in disease [4]. *sarA *itself is regulated by several loci that modulate its function. We recently identified a novel gene, *msa*, that modulate the function of *sarA *[5]. We showed that *msa *is essential for full expression of *sarA *and that mutation of *msa *affected the expression of several virulence factors in both *sarA*-dependent and *sarA*-independent manners [5]. Microarray analyses of the *msa *mutant show that Msa has a global effect on genes in *S. aureus *(unpublished data). These studies indicate that *msa *is an important locus in *S. aureus *and that the characterization of the Msa protein would be very useful in understanding staphylococcal regulatory networks.

### Computational tools

Several bioinformatics tools have been developed to predict the structure and functional properties of bio molecules. These tools use a wide variety of algorithms to predict the properties of proteins at different levels [6,7]. The accuracy of these bioinformatics tools has been improving; however, each tool has its own advantages and disadvantages. A particular algorithm has its own characteristic specificity, sensitivity, robustness, computational cost, etc. These characteristics can be tested against benchmarks of known datasets (e. g., Critical Assessment of Techniques for Protein Structure Prediction – CASP). In order to make the most accurate predictions, several methods should be used to build a consensus.

The aim of this work is to predict the structure and functional properties of the Msa protein of *S. aureus *to the highest possible accuracy. Our prediction results show that the Msa is a putative integral membrane protein with three probable transmembrane regions. We also predict that the Msa contains phosphorylation sites in the loop regions (both inside and outside the membrane). The 3-D structure analysis of the Msa also predicts the presence of putative binding sites. Thus, based on this computational analysis, and previous experimental data [5] we hypothesise that Msa might play a role in signal transduction. The fact that Msa has no known homolog means that it would be a novel signal transducer.

## Results and discussion

### Primary sequence analysis

The conceptually translated Msa protein is made of 133 amino acids with a predicted molecular weight of 15.6571 kDa and an isoelectric point (pI) of 6.71. The GRAVY index value 1.021 shows that Msa is probably an insoluble protein. The Codon adaptation index (CAI) value predicts the Msa as a highly expressed protein. This is consistent with experimental results described previously by our group [5].

### Homology and similarity

The Msa is highly conserved among the different strains of *S. aureus *(RF122, MRSA252, MSSA476, MW2, COL, Mu50, N315, and NCTC 8325). Even though there were several variations in the nucleotide sequences, we observed good conservation at the amino acid level. Multiple sequence alignment and phylogenetic analysis of both nucleotide sequences (SAUSA300_1294, SACOL1436, SAOUHSC_01402, SAV1401, *msa*, SAS1342, MW1289, SAR1413, and SAB1257c) and protein sequences (YP_493991, YP_186288, YP_499929, NP_371925, NP_374514, YP_043463, NP_646106) from different strains show that they are identical. The only two exceptions were strains RF122 and MRSA252 which showed slight variations in the Msa sequences. In RF122, the protein sequence (YP_416734) was 97% similar to the Msa sequence from N315 while in MRSA252, the protein sequence (YP_040815) was 98% similar to the Msa from N315. The phylogeny of the Msa protein closely resembled that of the phylogeny of these organisms as determined by Multi Locus Sequence Typing (MLST) [8]. The position and effect of mutations in the Msa protein sequence of the strains MRSA252 and RF122 are discussed in the "3-D structure prediction and analysis" section.

Our similarity search results against several sequence and structure databases, using different BLAST programs, showed that there were no significant closely related homologs for the Msa protein, except for one in *S. epidermidis*. Even though there were no significant (based on E-value and score) homolog for Msa, BLAST also listed several membrane proteins with remote similarities (alignment Score of 32–35 and E values scores from 0.91–10) only to the first few amino acids of the Msa protein (that corresponds to the predicted signal peptide region).

### Localization predictions

All the tools used to predict the cellular location of the protein indicated that Msa is a putative membrane protein. This prompted us to examine the sequence for presence of signal peptide and potential cleavage sites in the Msa protein sequence. Seven out of eight signal peptide prediction tools indicated the presence of a potential signal peptide in the Msa protein (Table 1). The majority of the programs also predicted an N-terminal cleavage site between the amino acid 19 and 20.

**Table 1:** Signal peptide and cleavage position prediction for the Msa protein

	**Signal Peptide**	**Cleavage Position**
**SignalP**	Present	29
**PrediSi**	Present	29
**sigcleave**	Present	20
**PSORT**	Present	20
**Phobius**	Present	20
**SIG-Pred**	Present*	20
**iPSORT**	Present	No prediction
**SOSUIsignal**	Absent	No prediction
**Consensus**	Present	20

* Predicted as an eukaryotic signal peptide

### Topology predictions

We performed topology analysis on the Msa sequence using several prediction programs that yielded widely discrepant results (Table 2). Even though most programs failed to recognize the signal peptide, a consensus topology emerged (Table 3). The predicted topology of the Msa is IN-OUT with three putative transmembrane segments (from amino acid positions 27–47, 54–75, 108–125). The N-terminal is predicted as present in the cytoplasmic side of the membrane while the C-terminal is predicted as outside the membrane. Our consensus topology also passed the positive-inside rule and charge bias test [9], with a charge bias of +1 towards the inside of the membrane.

**Table 2:** Topology predictions for the Msa protein

	**TopPred**	**TMpred**	**PHDhtm**	**TMHMM**	**SPLIT**	**HMMTOP**	**MEMSAT**	**DAS**	**TSEG**
**N-terminal**	IN	IN	OUT	IN	IN	IN	IN	-	-
**# of TMS**	3	3	3	4	4	4	4	4	4
TMS 1*	3–23	-	14–34	3–21	2–23	6–23	7–23	8–21	3–22
**TMS 2**	27–47	29–47	-	25–47	27–47	28–47	30–47	27–44	24–47
**TMS 3**	-	55–75	55–72	54–76	54–69	60–77	54–70	57–67**	54–75
**TMS 4**	106–126	107–123	108–125	108–125	107–126	108–125	108–125	110–124	105–128

TMS, transmembrane segments

* Analysis with several signal peptide prediction tools indicate that this TMS is a putative signal peptide

** Probability not significant

**Table 3:** Consensus topology for the Msa protein including the N-terminal signal peptide prediction

	**N-terminal**	**TMS 1**	**TMS 2**	**TMS 3**
**TopPred**	IN	27–47	-	106–126
**TMHMM**	IN	25–47	54–76	108–125
**TMpred**	IN	29–47	55–75	107–123
**SPLIT**	IN	27–47	54–69	107–126
**HMMTOP**	IN	28–47	60–77	108–125
**MEMSAT**	IN	30–47	54–70	108–125
**PHDhtm**	OUT	14–34	55–72	108–125
**TSEG**	No Prediction	24–47	54–75	105–128
**DAS**	No Prediction	27–44	54–67*	110–124
**Consensus**	IN	27–47	54–75	108–125

TMS, Transmembrane segment

* Probability not significant

**Figure 1:**
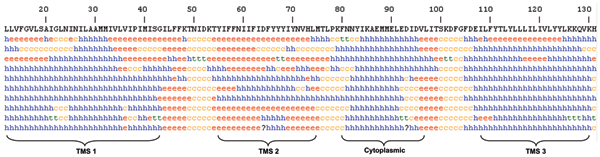
**Consensus secondary structure predictions for the Msa protein**. Three transmembrane segments (TMS) and a cytoplasmic helix are predicted.

Secondary structure prediction results indicated the presence of four distinct helical regions (Figure 1). One helical region corresponds to the cytoplasmic helix while the other three correspond to the integral membrane helices. These results are consistent with the predicted topology.

### Domains/patterns/motifs

We searched for the presence of domains, patterns and motifs in the Msa protein sequence, to gain insight into its functions and structure. The SMART results showed the presence of all the structural domains that we earlier identified using topology prediction programs and signal peptide prediction programs, viz. an N-terminal signal peptide and three transmembrane regions. In addition, SMART also predicted the presence of a PreATP-grasp domain (d1gsa_1) from the SCOP database. Even though this result had an E-value of 1.5, it was interesting because the predicted domain is a putative binding domain and falls in the predicted cytoplasmic region of Msa (residues 85–116). Our pattern search in the Msa protein sequence, using different programs against the PROSITE database, gave similar results (except for PPSearch, which did not predict the Tyrosine kinase site at position 48), showing the presence of three putative phosphorylation sites (Table 4). All of the predicted sites were found in the exposed regions of the Msa. Analysis of the location of these putative phosphorylation sites showed that two of the putative phosphorylation sites are outside the membrane while one of them is predicted in the cytoplasmic region. We also observed that these putative phosphorylation sites are highly conserved among different strains of *S. aureus*. This suggests that Msa might be phosphorylated by kinases in the cytoplasm as well as kinases on the outside of the membrane (e.g. from the host cells). These predictions further suggest that Msa might function as a signal transducer and provides important targets for mutagenesis experiments to test this hypothesis.

**Table 4:** Prosite patterns predicted in the Msa protein

	**Protein Kinase C**	**Casein Kinase II**	**Tyrosine Kinase**
**PPSearch**	99	49, 99	-
**PSITE**	99	49, 99	48
**ScanProsite**	99	49, 99	48
**Consensus**	99	49, 99	48

Numbers denote residue position in the Msa protein sequence

Membrane bound receptors are important components of signal transduction in all living systems. The major class of receptors in eukaryotes contain seven transmembrane segments (7 TM). Prokaryotes use 7 TM class receptors also, however, a recent study showed that prokaryotes carry novel receptor classes that have transmembrane segments ranging from one to eight [10,11]. The Msa protein sequence did not have significant homology with any of the known receptors and experimental studies are underway to evaluate its function as a signal transducer.

### 3-D structure prediction and analysis

**Figure 2:**
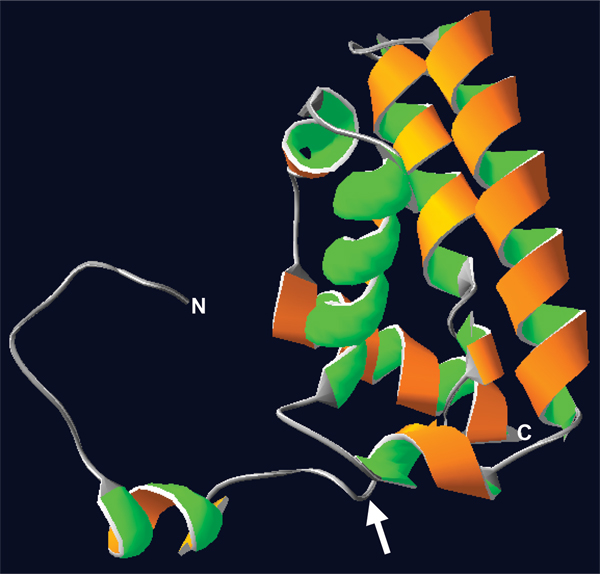
**Predicted tertiary structure of the Msa protein showing the three transmembrane helices**. Arrow indicates the predicted cleavage site for the putative signal peptide. N, N-terminus; C, C-terminus

**Figure 3:**
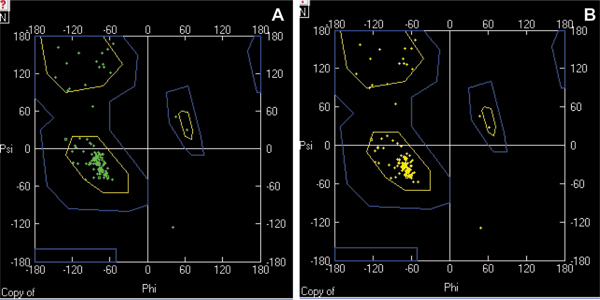
Ramachandran plot for the predicted tertiary structure of the Msa protein pre (A) and post (B) refinement.

Homology based tertiary structure prediction for the Msa protein failed, because of the lack of homologous structures. We used fold recognition based structure prediction server Phyre to model the tertiary structure of the Msa protein. Visualization and analysis of the predicted structure using Swiss-PDB Viewer (SPDBV) showed that the predicted structure correlated with the other predicted structural features of Msa in terms of the number and positions of the transmembrane helices (Figure 2). We refined the predicted structure by fixing side chains, fixing problematic loops, removal of amino acid clashes (bumps) and energy minimization. The refinements did not yield any drastic change in the initial predicted structure. This was confirmed by visually inspecting the structure and verifying the backbone structure using Ramachandran plot (Figure 3) and computing the total energy difference between the initial model and the refined model.

**Figure 4:**
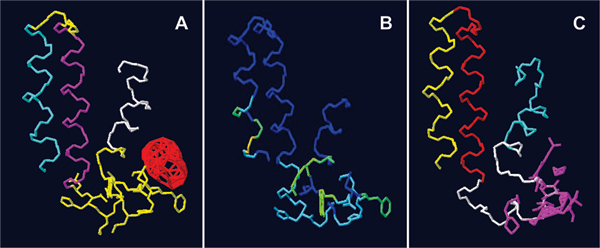
**Binding site predictions for the Msa protein**. (A) ProFunc predicted binding site (red); (B) PINUP predicted binding site (interface in green); (C) Q-SiteFinder predicted binding site and binding residues (pink)

We analysed the predicted tertiary structure for clefts and binding sites using ProFunc server and found putative binding sites in the cytoplasmic region between the second and the third transmembrane helices (Figure 4A). We also used PINUP to predict putative interface residues in the similar region (Figure 4B). Another binding site prediction server Q-SiteFinder also predicted similar binding site and binding site residues (Figure 4C).

ProFunc also predicted a "nest" near the putative phosphorylation site (residues 47–50) which was predicted outside the membrane [12]. The Msa has all the conserved residues that make up the predicted "nest". The predicted "nest" in Msa shows features of an anion-binding site. Such "nests" are characteristic functional motifs, which are found in ATP- or GTP binding proteins.

Multiple sequence alignment of the Msa protein sequence from 11 different strains of *S. aureus *revealed 12 mutations in strain RF122 and seven mutations in strain MRSA252 relative to consensus. Mutations at amino acid positions 111, 131 and 133 were found in both MRSA252 and RF122 strains. None of these mutations were found in the predicted phosphorylation sites, predicted signal peptide sites or in the predicted anion-binding "nest". But many of the mutations were found both in the integral membrane segments as well as in the other parts of the loop regions. Only one out of the 12 mutations had the replacement (functionally different amino acid), while others were substitutions (functionally similar amino acids), in the strain RF122. In the strain MRSA152, two out of seven mutations were replacements, while others were substitutions. MRSA strain had three mutations in the predicted pre-ATP grasp domain, out of which one had an amino acid replacement. RF122 strain had only one amino acid substitution in the pre-ATP grasp domain. This indicates that the predicted functional sites are constrained from mutation.

## Conclusion

**Figure 5:**
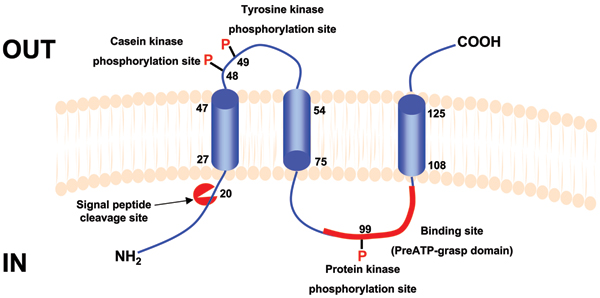
Predicted model for the Msa protein showing structural and functional features.

We predict that Msa is a membrane protein with a cleavable N-terminal signal peptide sequence, followed by three integral transmembrane regions. The Msa is also predicted to have an IN-OUT topology with at least two putative phosphorylation sites, one outside the membrane and one in the cytoplasmic region. A putative binding site is also predicted in the cytoplasmic region of Msa. Based on these predictions we put forward a model for the Msa protein (Figure 5). This model also prompted us to hypothesise that Msa might function as a novel signal transducer between the environment and the cytoplasm. This model will be used to design and execute experiments to confirm the functions and topology of Msa and further our understanding of its role in the pathogenesis of *S. aureus*.

## Methods

For a complete list of online tools used, see additional file 1.

### Primary sequence analysis

We used the protein sequence (Accession ID: NP_374514) obtained by conceptual translation of the *msa *open reading frame from the *S. aureus *N315 genome (NCBI database). The primary sequence analysis was performed using ProtParam, ProtScale [13] and SAPS [14]. ProtScale was used to predict the Msa profile based on several amino acid scales. ProtParam computes properties like molecular weight, theoretical pI, instability index and grand average of hydropathicity (GRAVY). SAPS predicts significant features of protein sequences like charge-clusters, hydrophobic regions, compositional domains etc.

### Similarity searching

Similarity searching was done using different programs at NCBI like BLASTP, PSI-BLAST [15] and CDART [16] against several different databases like NR, SWISSPROT and PDB. Multiple sequence alignment and phylogenetic analysis were done using Accelrys Gene v2.5 (Accelrys Inc., San Diego, CA).

### Sub-cellular localization

The Sub-cellular localization and the functional categorization of Msa were predicted using ProtFun 2.2 [17], PSORT [18], ProtCompB V-3 [19], PRED-CLASS [20] and SVMProt [21].

ProtFun uses *ab initio *methods to predict the cellular role category. PSORT uses a rule-based method to predict protein localization sites. ProtCompB combines several methods such as linear discriminant function-based predictions, direct comparison with homologous proteins of known localization, prediction of functional peptide sequences etc., to identify the sub-cellular localization of proteins. PRED-CLASS uses cascading neural networks to classify proteins in to different classes like membrane, globular, fibrous and mixed. SVMProt uses a support vector machine based approach to functionally classify protein sequences.

### Signal peptide prediction

Signal peptide prediction was done using SignalP [22], PrediSi [23], sigcleave [24], PSORT [18], Phobius [25], SIG-Pred [26], SOSUIsignal [27] and iPSORT [28].

SignalP 3.0 uses artificial neural networks and hidden Markov models to predict signal peptides and their cleavage sites. PrediSi predicts signal peptide sequences and their cleavage sites based on a position weight matrix that also takes into consideration the amino acid bias present in the proteins. Sigcleave is one of the early tools to predict the signal cleavage sites based on weight matrices. Sigcleave is distributed as part of the EMBOSS package. Phobius is a combined transmembrane protein topology and signal peptide predictor that uses a well trained hidden Markov model. SIG-Pred predicts signal peptides and their cleavage position based on weight matrices. SOSUIsignal uses a high performance system to predict signal peptides, using a three module software system that recognises the three-domain structure of signal peptides. iPSORT predicts the signal peptides based on a rule based system.

### Topology prediction

The Topology of Msa protein was predicted using TopPred [29], TMpred [30], PHDhtm [31], TMHMM [32], SPLIT [33], HMMTOP [34], MEMSAT [35], DAS [36] and TSEG [37]. We also computed the charge bias of the generated models, based on the positive-inside rule [9].

TopPred II predicts the topology of a protein based on its hydrophobicity profile and positive-inside rule. TMpred algorithm is based on the statistical analysis of TMbase, a database of naturally occurring transmembrane proteins, using a combination of several weight-matrices for scoring. PHDhtm uses a neural network based approach with the evolutionary information to predict the locations of the transmembrane helices. TMHMM predicts transmembrane regions based on the hidden Markov model. SPLIT 4.0 predicts location of transmembrane helices by performing an automatic selection of optimal amino acid attribute and corresponding preference functions. HMMTOP 2.0 prediction is based on the hypothesis that the difference in the amino acid distributions in various structural parts determines the localization of the transmembrane segments. MEMSAT applies a novel dynamic programming algorithm to recognize membrane topology models by expectation maximization. DAS uses dense alignment surface method to predict transmembrane regions. TSEG uses a discriminant function to predict the transmembrane segments.

### Secondary structure prediction

We used the NPS (Network Protein Sequence Analysis) consensus secondary structure server [38]. This server runs the input sequence against several different secondary structure prediction tools and generates a consensus secondary structure out of them.

### Domains/patterns/motifs prediction

SMART (Simple Modular Architecture Research Tool) [39] was used to identify the presence of any domains in the Msa protein. We used different pattern searching applications (PPSearch [40], PSITE [41] and ScanProsite [42]), that use PROSITE [43] database, to predict functionally relevant patterns in Msa protein.

### 3-D Structure prediction and analysis

Initial attempts to predict the tertiary structure of Msa were done using different approaches like homology modelling, threading and *ab initio*. Automated homology modelling servers Swiss-Model [44] and ModWeb [45] were used for homology modelling. Predictions described in this study were done using fold recognition tools 123D+ [46], GenThreader [47], a new version of 3-D PSSM (Phyre) [48].

The quality of the predicted structure was examined using an online version of the WHATIF [49] program. Structure refinement was done using both WHATIF and Swiss-PDB Viewer [50]. Structure visualization was done using Swiss-PDB Viewer.

The 3-D structure of the Msa protein was analysed for clefts and binding surfaces using ProFunc [51], Q-SiteFinder [52], PINUP [53] and SuMo [54].

### Meta servers

We also used meta servers like SCRATCH [55], ProSAL [56] and MetaPP [57] for predicting structure and functional properties of the Msa protein.

## Competing interests

The authors declare that they have no competing interests.

## Authors' contributions

VN did the analysis and drafted the manuscript. MOE directed the whole research and critically revised the manuscript.

## Supplementary Material

Additional file 1 **URL's of the tools and applications used in this study**. URL's of the tools and applications used in this study. This file contains the list of URL's of all the online tools used to predict the structure and functional aspects of Msa protein. Click here for file
